# CO_2_‐Induced Spin‐Lattice Coupling for Strong Magnetoelectric Materials

**DOI:** 10.1002/advs.202303692

**Published:** 2023-11-16

**Authors:** Bo Gao, Song Xu, Qun Xu

**Affiliations:** ^1^ College of Materials Science & Engineering Zhengzhou University Zhengzhou 450001 P. R. China; ^2^ Institute of Advanced Technology Zhengzhou University Zhengzhou 450001 P. R. China

**Keywords:** biaxial strain, magnetoelectric nanomaterials, nanocomposites, self‐assembly, spin‐lattice coupling

## Abstract

The preparation of 2D magnetoelectric (ME) nanomaterials with strong ME coupling is crucial for the fast reading and writing processes in the next generation of storage devices. Herein, 2D BaTiO_3_ (BTO)‐CoFe_2_O_4_ (CFO) ME nanocomposites are prepared through a substrate‐free coupling strategy using supercritical CO_2_. Such 2D BTO‐CFO with strong coupling is built through alternating in‐plane and out‐of‐plane epitaxy stacking, leading to remarkable mutual biaxial strain effects for spin‐lattice coupling. As a results, such strain effect significantly enhances the ferroelectricity of BTO and the ferrimagnetism of CFO, where an unexceptionally high ME coupling coefficient of (325.8 mV cm^−1^ Oe^−1^) is obtained for the BTO‐CFO nanocomposites.

## Graphical abstract

2D BaTiO_3_ (BTO)‐CoFe_2_O_4_ (CFO) nanocomposites are prepared by a substrate‐free coupling strategy using supercritical CO_2_, which constructs nanocomposites by alternative in‐plane and out‐of‐plane epitaxial stacking. The stacking generates spin‐lattice coupling with mutual biaxial strain, which greatly enhances the ferroelectricity and ferrimagnetism of BTO and CFO, respectively. As a result, unexceptional strong magnetoelectric coupling is achieved for BTO‐CFO.

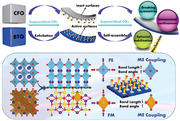

## 1 Introduction

With the emergence of next‐generation data storage devices, materials with higher reliability, better energy efficiency, and faster data transfer speed are urgently demanded. By adjusting the magnetization state, magnetic materials have been applied to write data in magnetic random‐access memory (MRAM). However, this technology suffers from high energy consumption, overheating, and low writing speed.^[^ ^1^ ^]^ To improve the writing speed, utilizing ferroelectric random‐access memory (FeRAM) devices instead of conventional MRAM has been proposed to be a feasible strategy.^[^ ^2^ ^]^ Despite the rapid write speed, the reading rate of FeRAM devices remains sluggish.^[^ ^3^ ^]^ Thus, multiferroic materials coupled with ferroelectric and ferromagnetic sequences are expected to achieve rapid hybrid read‐write operations, which makes them appealing for high‐performance data storage devices.^[^ ^4^ ^]^ To date, single‐phase multiferroic materials are extremely rare, with limited magnetoelectric (ME) coupling.^[^ ^5^ ^]^ In contrast to the single‐phase multiferroic materials, multi‐phased composites with indirect ME effect provide strong ME coupling at ambient temperature.^[^ ^6^ ^]^

Spin‐lattice coupling, an effective strategy that breaks inversion and time‐reversal symmetry, is proposed as one of the most effective strategies to modulate the electronic structure, leading to novel ferromagnetic and ferroelectric properties. For example, spin‐lattice coupling from strain engineering has been reported to increase superconductivity,^[^ ^7^ ^]^ ferromagnetism,^[^ ^8^ ^]^ ferroelectricity,^[^ ^9^ ^]^ and ME coupling.^[^ ^10^ ^]^ Noteworthy, experimental, and theoretical results suggest that strain engineering can effectively increase the ferroelectricity of BaTiO_3_ (BTO)^[^ ^11^ ^]^ and the ferrimagnetism of CoFe_2_O_4_ (CFO).^[^ ^12^ ^]^ Thus, constructing BTO‐CFO nanocomposites through strain engineering is proposed to be an attractive strategy to couple ferrimagnetism/ferroelectricity, leading to composites with strong ME coupling. In this paper, 2D nanocomposites coupled with BTO and CFO were obtained through alternative in‐plane and out‐of‐plane self‐assemble growth of 2D BTO and CFO nanosheets using supercritical CO_2_ (SC CO_2_) treatment, which achieves epitaxial stabilization, dimensional confinement and symmetry breaking of the BTO‐CFO nanocomposites simultaneously.^[^ ^13^ ^]^ The coupling between BTO and CFO leads to a certain biaxial strain in each component, which enhances ferroelectricity in BTO and ferrimagnetism in CFO through spin‐lattice coupling. As a result, due to the presence of lattice strain, the coupling between BTO and CFO exhibits the unexceptional high coupling coefficient for BTO‐CFO material (348.5 mV cm^−1^ Oe^−1^). In addition to a nanocomposite with a high ME coupling coefficient, we anticipate this work provides a novel strategy to prepare multiferroic materials for next‐generation data storage devices.

## 2 Microstructural Analysis

**Figure 1:**
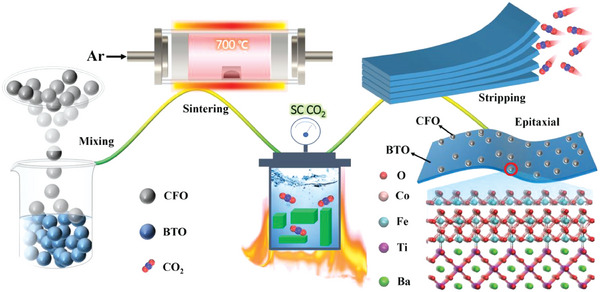
Flow chart for the preparation of BTO‐CFO nanocomposites.

The general preparation of BTO‐CFO nanocomposites is shown in **Figure** **1**, with a detailed synthetic protocol given in the Experimental Section. BTO and CFO mixed at molar ratios of 1:0.1, 1:0.2, 1:0.5, and 1:1 were abbreviated to BTO‐0.1CFO, BTO‐0.2CFO, BTO‐0.5CFO, and BTO‐1CFO, respectively.

**Figure 2:**
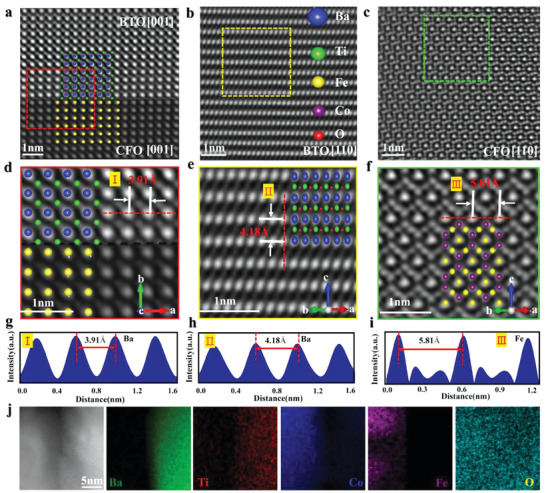
Microstructural characterization of nanosheets. a) Nanocomposite structure under high‐angle annular dark‐field scanning transmission electron microscope. The interface between the BTO and CFO (001) plane. b) BTO lattice structure along the [110] direction. c) CFO lattice structure along the [110] direction. d) Locally enlarged view of the nanocomposite interface. e) Local enlargement of the BTO lattice structure along the [110] direction and the corresponding atomic structure. f) Local enlargement of the CFO lattice structure along the [110] direction and the corresponding atomic structure. g–i) Correspond to the intensity distribution of the atomic peaks along the red dashed lines in (d), (e), and (f), respectively. The distance between two adjacent peaks corresponds to the spacing between two adjacent atoms. j) The energy dispersive spectroscopy (EDS) mapping images of BTO‐CFO.

High‐angle annular dark field scanning transmission electron microscopy (HAADF‐STEM) is used to investigate the morphology of the nanostructures, where a relatively clear lattice structure and a distinct interface are characterized (**Figure** **2**a). In addition to the (001) plane of BTO and CFO shown in Figure 2a, the (110) plane of single‐phase BTO and single‐phase CFO were shown in Figure 2b,c. According to Figure 2d,g atomic spacing for Ba atoms is proposed to be 3.91 Å along the *a*‐axis direction on the (001) plane, which is 2.13% smaller than the a‐lattice constant (3.995 Å), indicating significant compression of BTO along the equatorial direction of the octahedron. The lattice structure of the BTO (110) plane is shown in Figure 2b, where structural distortion was found (Figure 2e). The atomic spacing of Ba along the *c*‐axis direction on the (110) plane is 4.18 Å (Figure 2h), which is 3.64% larger than the c‐lattice constant (4.033 Å). It indicates that the BTO is stretched along the octahedral axial direction. Overall, biaxial strain in the BTO is confirmed by the stretching and compression along the axial and equatorial direction of the octahedron, respectively. In addition to BTO, the lattice structure of the CFO along the (110) direction is given in Figure 2c,f,i, where the spacing of the Fe atom is reduced to 5.81  from 5.93 Å, which is a typical value for crystalline space of (220) plane of CFO.^[^ ^14^ ^]^ Such atomic spacing reduction indicates the compression of CFO along the axial direction. Finally, the effective coupling between BTO and CFO is confirmed by energy dispersive spectroscopy (EDS) mapping images (Figure 2j).

Additionally, bright field TEM characterization was utilized to reveal further morphological information of BTO‐CFO. The average lateral size of the nanosheets is decreased from 50.95 to 34.65 nm, as the CFO content increases (Figure S1, Supporting Information). Since the critical single‐domain size of CFO is ≈40 nm,^[^ ^15^ ^]^ BTO‐0.1CFO and BTO‐0.2CFO, whose size is in the range of 40–50 nm, are proposed to be multi‐domain structures. In contrast, since the size of BTO‐0.5CFO and BTO‐1CFO are in the range of 30–40 nm, exhibit single‐domain structure. The conversion from multi‐ to single‐domain structure is further confirmed by magnetic force microscopy (MFM) (Figure S2, Supporting Information). Additionally, a clear interface between the two phases can be seen in Figure S3 (Supporting Information), but the atomic structure cannot be clearly seen in the right half, presumably due to the height difference between the BTO and CFO phases. To confirm the thickness of the BFO and CFO layer, atomic force microscopy (AFM) was conducted, where the thickness of the 2D nanosheets is characterized to be ≈10–12 nm (Figure S4, Supporting Information). This value is far larger than the result (4.5 nm) obtained from the treatment of pure BTO by SC CO_2_ in our previous work.^[^ ^16^ ^]^ The thickness difference is rationalized by dative epitaxy,^[^ ^17^ ^]^ where the BTO layer is directly coupled to a CFO layer instead of another BTO layer. Based on the variation of the sample height, the thickness of the BTO and CFO layers are estimated to be ≈6 and 4 nm, respectively.

**Figure 3:**
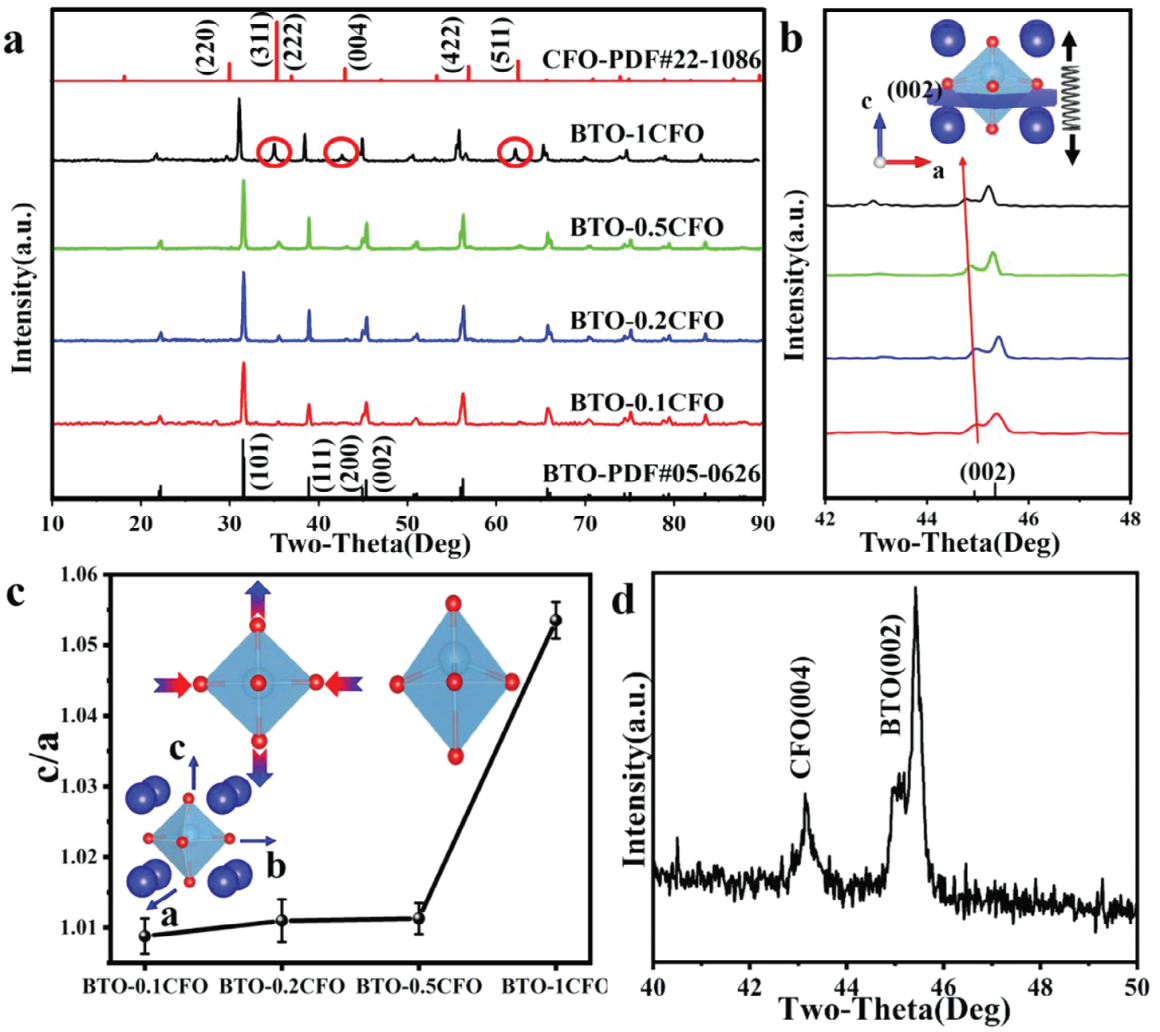
Characterization of the nanosheets. a) Accurate lattice structure analysis of BTO‐CFO samples by XRD. b) Magnification of (002) peak of BTO‐CFO nanocomposites. c) Variation of the c/a value of BTO obtained by XRD refinement with increasing CFO content. d) XRD patterns for locally magnified (40°−50°) of BTO‐1CFO sample.

X‐ray diffraction (XRD) (**Figure** **3**a) was utilized to characterize the lattice structure of BTO‐CFO. The (002/200) peak at 2θ = 45° indicates a tetragonal distortion (JCPSB No. 05–0626), corresponding to the ferroelectric state of BTO.^[^ ^18^ ^]^ Such peak splitting is presumably due to the electrostatic repulsion between the 3d electrons of Ti^4+^ and the 2p electrons of O^2−^, which exhibit a distorted perovskite lattice with tetragonal geometry. It's worth noting that as the amount of CFO increases, the major peak (101) of BTO approaches the peak position (220) of CFO (JCPSB No. 22–1086),^[^ ^19^ ^]^ which can be rationalized by the two‐phase interactions. The peak position of the BTO (002) in Figure 3b is shifted toward a lower diffraction angle, consistent with the presence of tensile strain.^[^ ^20^ ^]^ The increase in the crystallographic spacing of the (002) plane corresponds to the stretching of the BTO along the *c*‐axis (inset of Figure 3b). Meanwhile, XRD refinement (Figure S5, Supporting Information) confirmed the significant increase of c/a (1.0088–1.0535) concomitant with the growth of CFO content (Figure 3c; Table S2, Supporting Information), which is the result of a combination of compression in the equatorial plane of the octahedron and stretching in the axial plane (inset of Figure 3c). This value is much greater than conventional tetragonal BTO. Noteworthy, peaks corresponding to CFO were observed for all samples (red circles in the figure), whose intensity increases as the growth of CFO content, consistent with the formation of BTO‐CFO nanocomposites. According to the XRD patterns for locally magnified (40°−50°) of BTO‐1CFO sample (Figure 3d), the peak positions of CFO (004) and BTO (002) are consistent with the epitaxially grown BTO‐CFO.^[^ ^21^ ^]^

The optical modes at 179, 306, 512, and 716 cm^−1^ are observed for BTO‐CFO according to Raman spectra (Figure S6, Supporting Information), consistent with tetragonal P4mm crystal symmetry.^[^ ^22^ ^]^ The vibrational peak at 179 cm^−1^ is attributed to the asymmetric metal‐oxygen vibrations. Due to the coupling between CFO and BTO, lattice disorder accounts for the broad spectrum in the 200–275 cm^−1^ range.^[^ ^23^ ^]^ The symmetric stretching of TiO_6_ octahedra is represented by the transverse mode at 512 cm^−1^.^[^ ^24^ ^]^ Meanwhile, characteristic peaks corresponding to CFO in BTO‐1CFO are observed. The T_2g_ modes at 209 cm^−1^ and 471 cm^−1^ correspond to the translational motion of the TiO_6_ unit toward the A‐position and the motion of the oxygen atom coordinated to Fe^3+^, respectively.^[^ ^25^ ^]^ The vibrational peaks at 691 and 613 cm^−1^ for A_1g_ are attributed to the symmetric breathing modes of the FeO_4_ and CoO_4_ units, respectively.^[^ ^25^ ^]^ The Raman spectra further illustrate the effective coupling of the BTO and CFO, and the strain caused by the interaction of them.

## 3 ME Measurements

**Figure 4:**
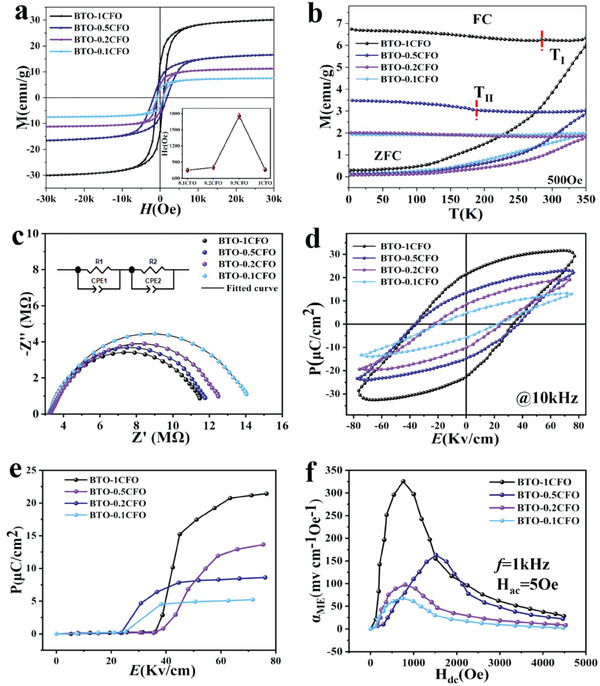
Ferrimagnetic and ferroelectric measurements. a) M−H curves at 300 K. Inset: Curve of coercivity (Hc) with CFO content. b) FC‐ZFC magnetization curve in an external magnetic field of 500 Oe. c) Combined Nyquist plots and their equivalent circuit measured. d) P–E loops at room temperature. e) Pr of the samples as a function of the electric field extracted from the PUND test. f) Magneto‐electric voltage coefficient as a function of dc magnetic field for the samples.

The ferroelectric and magnetic properties of the BTO‐CFO nanocomposite thin films were studied to evaluate the ME coupling. The magnetic hysteresis loop of BTO‐1CFO exhibits a saturation magnetization (Ms) of 30.11 emu g^−1^ at 300 K, with coercivity (Hc) and remanent magnetization (Mr) in the range of 1–2 kOe and 3.6–12.9 emu g^−1^, respectively (**Figure** **4**a). In addition, Table S3 and Figure S7 (Supporting Information) give data on Ms obtained by normalizing the CFO content, which is close to bulk CFO, Additionally, the Ms increases as the CFO content increases, indicating effective coupling between BTO and CFO can be achieved. Such coupling is expected to introduce strain on the CFO layer, which effectively improves its magnetic properties.^[^ ^12^ ^]^ In addition to CFO, such strain effect is also expected to enhance the magnetism of BTO, as proposed by our previous work.^[^ ^16^ ^]^

Furthermore, the Hc of BTO‐CFO nanocomposites increased from BTO‐0.1CFO to BTO‐0.5CFO, which decreases from BTO‐0.5CFO to BTO‐1CFO (inset of Figure 4a). This is rationalized by the transition of the BTO‐CFO nanosheets from multi‐domain to single‐domain, which has been confirmed by the structural characterizations. Generally, Hc generally depends on the size of the nanosheet. When the size of crystals is large enough (multi‐domain), the value of coercivity increases as the size of the nanosheet decreases.^[^ ^26^ ^]^ For single‐domain magnetic nanosheets, spontaneous magnetization occurs when the temperature is below the Curie or Néel temperature, and the magnetic moment is directed toward a certain crystallographic axis associated with the anisotropy energy (*E*_A_) of the magnetic crystal. The correlation between *E*_A_ and the volume (*V*) of the nanocrystal can be expressed in terms of Stoner‐Wohlfarth theory: *E*_A_ *= KV*sin^2^θ, (where *K* is the anisotropy constant of the magnetic crystal and θ is the angle between the easy axis and the magnetization direction).^[^ ^27^ ^]^ *E*_A_ is the energy barrier for the change of magnetization direction. Therefore, *E*_A_ decreases as the size of the nanosheet drops, which reduces the magnetic field required for the change of the moment direction (lower levels of Hc). The movement and rotation of the magnetic domain walls becomes easier and stronger ME coupling effects can be induced.^[^ ^28^ ^]^ With the help of CO_2_, CFOs are effectively coupled with BTO, where the CFO content modulates Hc by grain size regulation for strong ME coupling.

Zero‐field‐cooled (ZFC) and field‐cooled (FC) signal tests were carried out with an applied magnetic field H = 500 Oe (Figure 4b). In the ZFC curve, the magnetization increases under elevated temperatures due to the release of frozen spins with increasing thermal energy. At room temperature, no fluctuations in the magnetization intensity exist, which means that the magnetic domain is strongly coupled to resist thermal perturbations for strong ferrimagnetism.^[^ ^29^ ^]^ The magnetization of the FC curve increases as the temperature decreases, accompanied by slight fluctuations (marked as T_I_ and T_II_). According to literature precedents,^[^ ^30^ ^]^ BTO undergoes two structural transitions (tetragonal‐orthogonal, orthogonal‐diagonal) below 350 K. The crystal domain switching induced by the phase transition alters the local strain in CFO, which changes the magnetization. Therefore, the fluctuations in the FC curve are attributed to the BTO phase transition. In addition, the ZFC and FC curves split below 350 K, which confirms that the Curie temperature of the sample is higher than room temperature. This magnetic behavior is consistent with other CFO systems.^[^ ^31^ ^]^

To understand the electrical conductivity of the sample, the interrelationship between the real and imaginary parts of the impedance of BTO‐CFO was plotted (Figure 4c). The plots for all BTO‐CFO nanocomposites show a single circular arc, indicating that only grain effects are present for all structures.^[^ ^32^ ^]^ As the CFO content increases, the radius of the semicircle decreases, indicating a decrease in resistance.^[^ ^33^ ^]^ The overall higher impedance value reduces the leakage current and thus enhances polarization. In addition, leakage current measurements confirmed that the prepared samples have a small leakage current density (Figure S8, Supporting Information), which is expected to facilitate the polarization of BTO‐CFO nanocomposites.

The polarization versus electric field (P‐E) hysteresis of BTO‐CFO was measured at room temperature with a testing frequency of 10 kHz, where ferroelectricity hysteresis loops were observed for all of the samples (Figure 4d). In order to eliminate the effects of leakage, we conducted a positive‐up−negative‐down (PUND) test for the BTO‐CFO nanocomposites. This measurement can eliminate the contribution of nonferroelectric artifacts in the polarization amplitude, and the principle is shown in Figure S9 (Supporting Information). As shown in Figure 4e, the results indicate that the reversible polarization of the sample is close to the residual polarization measured by the hysteresis loop. This strongly confirms the authenticity of the polarization in the sample. For BTO‐1CFO, the spontaneous polarization (Ps) value, residual polarization (Pr), and coercivity (Ec) are characterized as 28.61 µC cm^−2^, 21.47 µC cm^−2^, and 36.01 kV cm^−1^, respectively. These ferroelectric polarization measurements increase with the growth of CFO content, which is higher than previously reported CFO materials.^[^ ^34^ ^]^ As the grain size decreases, the nanocomposite interface increases, which significantly enhances the strain and the pinning of domain wall motion. As a result, the coercivity field is significantly enhanced. Additionally, the Ps data were normalized according to the BTO content (Table S4, Figure S10, Supporting Information), which increases under higher CFO content. This suggests that CFO is involved in ferroelectric polarization effects. The analysis of ferrimagnetism and ferroelectricity illustrates that ME effects are induced in both BTO and CFO under biaxial strain, which greatly improves the ME coefficient of BTO‐CFO. Furthermore, switching polarization (Psw) was measured by PUND (Figure S11, Supporting Information) and all samples have excellent endurance of > 10^8^ cycling.

The ME voltage coefficient α_ME_ of the BTO‐CFO nanocomposites was calculated using the following equation:

(1) $$\alpha_{\mathrm{ME}}=V_{\mathrm{out}}/\left(t\times H_{\mathrm{ac}}\right)$$

where t is the sample thickness and V_out_ is the induced voltage. The sample is biased with an ac magnetic field (Hac = 5 Oe) at frequency = 1 kHz, while a dc magnetic field Hdc is applied parallel to it. As shown in Figure 4f, the initial elevation of α_ME_ output can be attributed to the enhancement of elastic interaction between the two phases. The dc bias field H_dc_ with the maximum ME coefficient is close to the field at which the maximum magnetization. As H_dc_ increases, the deformation decreases leading to a decrease in the α_ME_ value. The maximum value of α_ME_ was measured to be 325.8 mV cm^−1^ Oe^−1^, much higher than the most of literature reported values (Table S5, Supporting Information). In addition, the temperature dependence of the ME coefficient for BTO‐1CFO is shown in Figure S12 (Supporting Information), where the maximum ME coefficient is observed at 350 K, which indicates that the ferroelectric polarization due to the deformation caused by magnetostriction reaches a maximum value at this temperature. The magnetic field‐dependent polarization curves (Figure S13, Supporting Information) show a tendency to enhance the ferroelectric polarization with increasing magnetic field.

## 4 DFT Calculation

**Figure 5:**
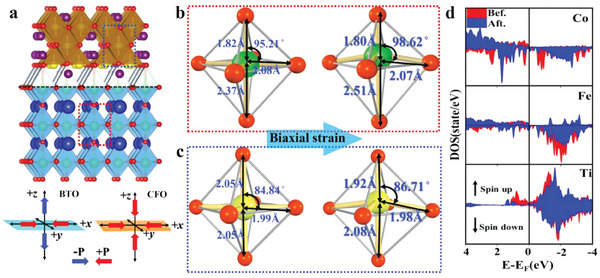
DFT calculations for ferrimagnetism and ferroelectricity. a) BTO‐CFO structure established from experiments. Down: Stretch and compression diagram for BTO and CFO. b) Variation of bond lengths and bond angles of BTO in the BTO‐CFO nanostructure before and after optimization. c) Variation of bond lengths and bond angles of CFO in the BTO‐CFO nanostructure before and after optimization. (To ensure reasonableness, we have chosen six layers of atoms in each of the two phases to form a nanostructure for the calculation). d) Comparison of the changes in the LDOS of Co, Fe, and Ti before and after the lattice structure change. (Red corresponds to before the lattice change, blue corresponds to after the lattice change, and the up and down arrows indicate spin‐majority and spin‐minority respectively).

BTO‐CFO nanostructure was constructed based on experimental results (**Figure** **5**a), where first‐principle density functional theory (DFT) was applied to investigate the interaction between the BTO and CFO components. For BTO, elongation of the Ti─O bond along the octahedral axis (from 2.37 to 2.51 Å) and compression within the equatorial plane (from 2.08 to 2.07 Å) were observed (Figure 5b). Such distortion is accompanied with the growth of O─Ti─O bond angle (95.21°–98.62°) (Figure 5b), consistent with the experimental observations. Meanwhile, the c/a value increases as CFO content grows, leading to a more tetragonal distorted BTO. According to the analysis of bond lengths and angles of Fe─O octahedra in CFO, it can be concluded that the Fe─O bond lengths are compressed along the octahedral axis (from 2.05 to 1.92 Å) and equatorial plane (from 1.99 to 1.98 Å) (Figure 5c), leading to a significant CFO distortion. Such distortion is consistent with experimental observations, where the interaction between BTO and CFO leads to a certain biaxial strain on each component and distorts the nanostructure.

The stretching and compression of the lattice discussed above are expected to affect the spin state of the electron according to the spin‐lattice coupling effect.^[^ ^35^ ^]^ Thus, the spin‐charge density distribution after the two‐phase coupling was calculated (Figure S14, Supporting Information), with the magnetic moments of the Fe and Co atoms at the interface are listed in Figure S15 (Supporting Information). Calculation suggests the magnetic moment is significantly higher at the interface, presumably due to lattice strain in the CFO. Figure 5d shows the local density of states (LDOS) of Fe, Co, and Ti atoms over the formation of BTO‐CFO nanostructure, in which increased asymmetry of spin‐majority and spin‐minority states of Fe and Co is observed after the formation of nanostructures, indicating the enhancement of spin polarization, which corresponds to the increase of the magnetic moment. This suggests that the distortion of the lattice will change the spin states of the electrons, ultimately leading to an increase in the magnetic moment, which is consistent with the experimental results that the strain from two‐phase coupling leads to an enhancement of the magnetism. In addition, the electron localization function at the black dashed line in Figure 5a suggests stronger Ti─O hybridization from the BTO‐CFO interaction of the nanostructure (Figure S16, Supporting Information). The corresponding partial density of states (PDOS) (Figure S17, Supporting Information) attributes the enhanced polarization intensity to the higher overlap between Ti─d and O─p orbitals and greater amplitude resonance. The enhancement of spin polarization atomic hybridization will contribute to the increase in polarization value (Table S6, Supporting Information). The above analysis illustrates that biaxial strain induces lattice stretching and compression after the formation of the BTO‐CFO nanostructure. This not only effectively enhances the atomic magnetic moment at the interface, but also induces enhanced ferroelectric polarization.

## 5 Conclusion

In summary, 2D multiferroic BTO‐CFO nanocomposites with strong ME coupling were prepared by an SC CO_2_‐assisted substrate‐free assembling strategy. Experimental and theoretical investigations suggest a significant strain effect exists over the coupling process, leading to spin‐lattice coupling that breaks inversion and time‐reversal symmetry to enhance the ferroelectricity and ferrimagnetism for ME coupling. Specifically, BTO‐CFO nanocomposites are formed by alternative self‐assembling growth over the SC CO_2_ treatment, which simultaneously breaks the symmetry and promotes ferrimagnetism/ferroelectricity coupling. Experimental results suggest the thickness and lateral dimensions of the BTO‐CFO nanocomposites decreases as the CFO content increase, which facilitates the interaction between the components. Such interaction leads to a certain biaxial strain on each other, which largely enhances the ferroelectricity of the BTO and the ferrimagnetism of the CFO. As a results, an unexceptional high ME coupling coefficient (325.8 mV cm^−1^ Oe^−1^) was characterized for BTO‐1CFO. Importantly, this work demonstrates a novel idea for the preparation of multiferroic nanocomposites, which modulate the ME coupling through spin‐lattice coupling using biaxial‐strain.

## 6 Experimental Section

### Sample Preparation

BTO has a tetragonal perovskite structure with lattice parameters a = 3.995, c = 4.033, and c/a = 1.009 (space group P4mm; NO.99).^[^ ^36^ ^]^ CFO was a ferrimagnet with an inverse‐spinel structure (space group Fd3m; No. 227).^[^ ^14^ ^]^ Oxide anions were arranged in a closely arranged cubic lattice and metal cations were surrounded by oxygen in the form of tetrahedrons or octahedrons. BTO‐CFO nanocomposites were prepared by a combination of solid‐phase sintering and supercritical CO_2_‐directed epitaxial growth strategy, the comprehensive fabrication procedure was presented below.

### Solid State Sintering

BTO and CFO were mixed according to the different molar ratios (1:0.1, 1:0.2, 1:0.5, and 1:1), which were defined as BTO‐0.1CFO, BTO‐0.2CFO, BTO‐0.5CFO, and BTO‐1CFO respectively. Then an appropriate amount of water was added to the BTO‐CFO mixture and ultrasonicated for 1 h to make a homogeneous suspension, which was dried in an oven at 200 °C for 4 h. Subsequently, the powder was put in a tubular furnace and heated to 700 °C at the heating rate of 5 °C per minute in Ar atmosphere for 1 h.

### Exfoliation

Powder (30 mg) was dispersed in 20 mL of ethanol/water solution (V_ethanol_:V_water_ = 1 : 1) and subjected to ultrasonic treatment for 4 h. The resulting suspension was labeled as ultrasonicated powder. Ultrasonicated powder suspension was directly transferred into the supercritical CO_2_ apparatus (a stainless‐steel autoclave with a heating jacket and a temperature controller). After the autoclave was heated to the designated temperature (120 °C), CO_2_ was charged into the reactor to 14 MPa and maintained for 4 h under continuous stirring. In this process, supercritical CO_2_ is used to facilitate the exfoliation and self‐assembly epitaxial growth of BTO and CFO. After CO_2_ was slowly released, the supernatant was collected by centrifugation at 3000 r.p.m. for 15 min. The reactor used in the experiment was custom‐made by Haian Research Instruments Ltd. (50 ml, 316 L stainless steel).

### Sample Preparation for Ferroelectric Measurements

The supernatant obtained by centrifugation was placed on Si/SiO2 substrate and dried in an oven at 65 °C for 5 h. Polymethyl methacrylate (PMMA) was spin‐coated on the surface of the substrate using spin coating, and the electrode area was then subjected to electron beam exposure. The substrate was immersed in a developer solution to remove the PMMA from the electrode region. 100 nm thick Au was vaporized at a deposition rate of 0.5 Å s^−1^ as the electrode. Excess PMMA was removed using acetone and blown dry with nitrogen.

### Characterizations

Transmission electron microscope (TEM) images were recorded on a FEI Tecnai G2 F20 at an acceleration voltage of 200 kV. The thickness of nanosheets was measured by an atomic force microscope (Bruker Dimension Icon). X‐ray diffraction (XRD) patterns were collected on a Bruker D8 Focus diffractometer (Bruker AXS, Germany) using Cu K radiation. Raman measurements were performed using LabRAM HR Evolution with laser wavelength of 633 nm. X‐ray photoelectron spectroscopy was performed using the AXIS Supra system. The magnetic measurement was carried out with a Physical Property Measurement System (quantum design, PPMS‐9). Ferroelectricity, leakage current, and PUND were characterized with a ferroelectric analyzer (America, Radiant Precision Multiferroic II). Temperature‐dependent ME response was measured in a superconducting magnet system (Multiferroic Magnetoelectric Measurement System – SuperME) using dynamic techniques. The ME‐induced AC voltage on the sample was collected by a lock‐in amplifier (Stanford Research SR830). The refinement of XRD data carried out using GSAS‐2 software and the results are shown in Figure S5 (Supporting Information), with more fitted parameters and phase fractions shown in Table S1 (Supporting Information).

### Calculation Method

The spin‐polarized density functional theory (DFT) calculations were performed using the Vienna Ab‐initio Simulation Package (VASP) code.^[^ ^37^ ^]^ The generalized gradient approximation (GGA) of the Perdew–Burke–Ernzerhof (PBE) functional with van der Waals correction was applied to optimize the geometric structures.^[^ ^38^ ^]^ The interactions between the ions and valence electrons were described by Projector augmented wave (PAW) potentials.^[^ ^39^ ^]^ A Monkhorst‐Pack k‐point grid of 4 × 4 × 4 was used for the geometric structure optimization and total energy calculations. The force on each atom was less than 0.01 eV Å^−1^, and the convergence criteria of the total energy for all the calculations were set as 1 × 10^−5^ eV. A plane wave cutoff energy of 450 eV was chosen for all of the calculations. In the course of the calculations, the surfaces are first cut and then a BTO‐CFO heterostructure is created. Since BTO‐CFO is a periodic structure, a 15 Å vacuum layer is created during the calculation in order to prevent the influence of the upper and lower surfaces. After the structure is optimized, other relevant properties are calculated.

### Statistical Analysis

The selected data are presented as the mean ± standard deviation. The transverse dimensions of the samples were counted using Image J software (Figure S1, Supporting Information).

## Conflict of Interest

The authors declare no conflict of interest.

## Supporting information

Supporting Information

## Data Availability

The data that support the findings of this study are available in the supplementary material of this article.
