# Supplementary material for: CO2‐Induced Spin‐Lattice Coupling for Strong Magnetoelectric Materials
Source: Adv Sci (Weinh). 2023 Nov 16;11(5):2303692. doi: 10.1002/advs.202303692 (PMC10837372; doi:10.1002/advs.202303692)
Supplement: Supplementary file 1 — Supporting Information [file ADVS-11-2303692-s001.pdf]

ADVANCED SCIENCE
Open Access

Supporting Information

for *Adv. Sci.*, DOI 10.1002/advs.202303692

CO_2-Induced Spin-Lattice Coupling for Strong Magnetoelectric Materials

*Bo Gao, Song Xu and Qun Xu**

Supporting Information

CO_2-Induced Spin-Lattice Coupling for Strong Magnetoelectric Materials

Bo Gao[1], Song Xu[2], Qun Xu[1,2]*

[1] College of Materials Science & Engineering, Zhengzhou University, Zhengzhou 450001, P.R.China.

[2] Henan Institute of Advanced Technology, Zhengzhou University, Zhengzhou 450001, P.R.China.

Table of Contents

Supplementary Figures S3

Supplementary Tables S16

References S18

Supplementary Figures

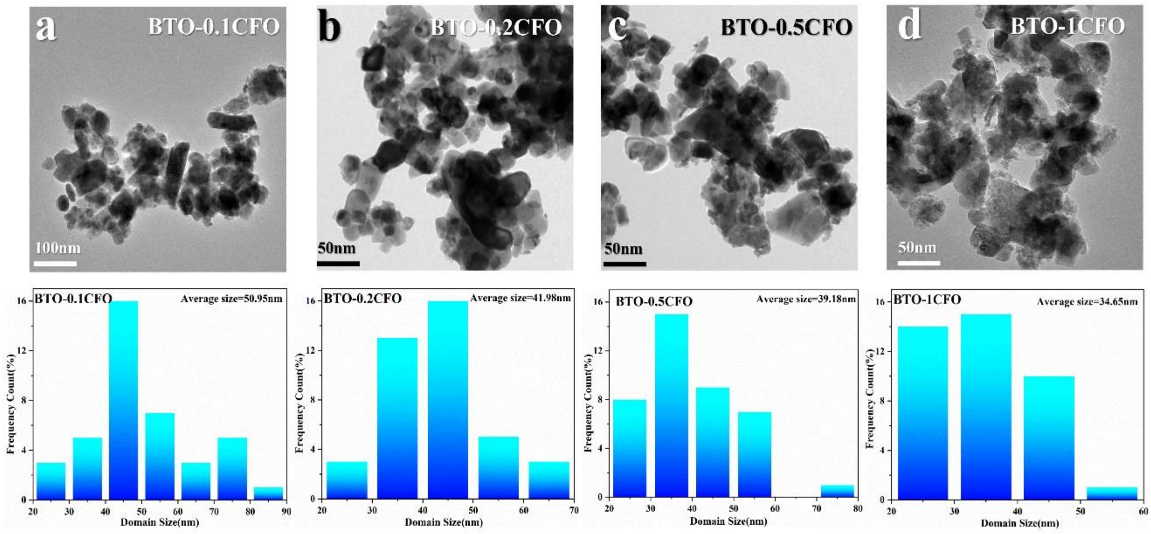

Fig. S1. Low magnification TEM images of the four structures. The histograms correspond to the scaled distribution of the lateral dimensions of the nanosheets.

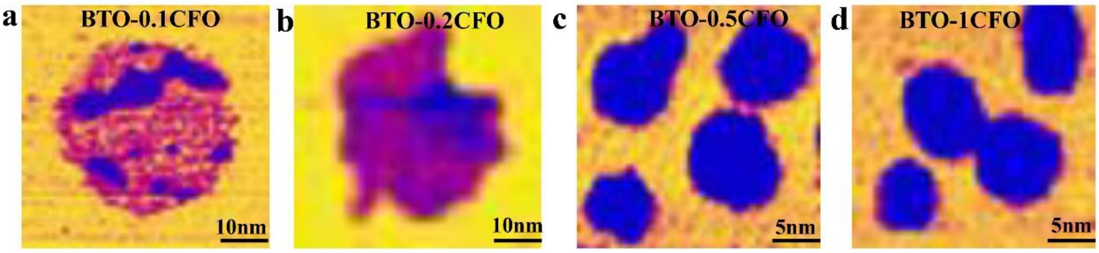

Fig. S2. Magnetic force microscopy (MFM) images of the four structures. The domain structure of BTO-0.5CFO and BTO-1CFO is single-domain, while BTO-0.1CFO and BTO-0.2CFO have a complex domain structure and should be recognized as multi-domain.

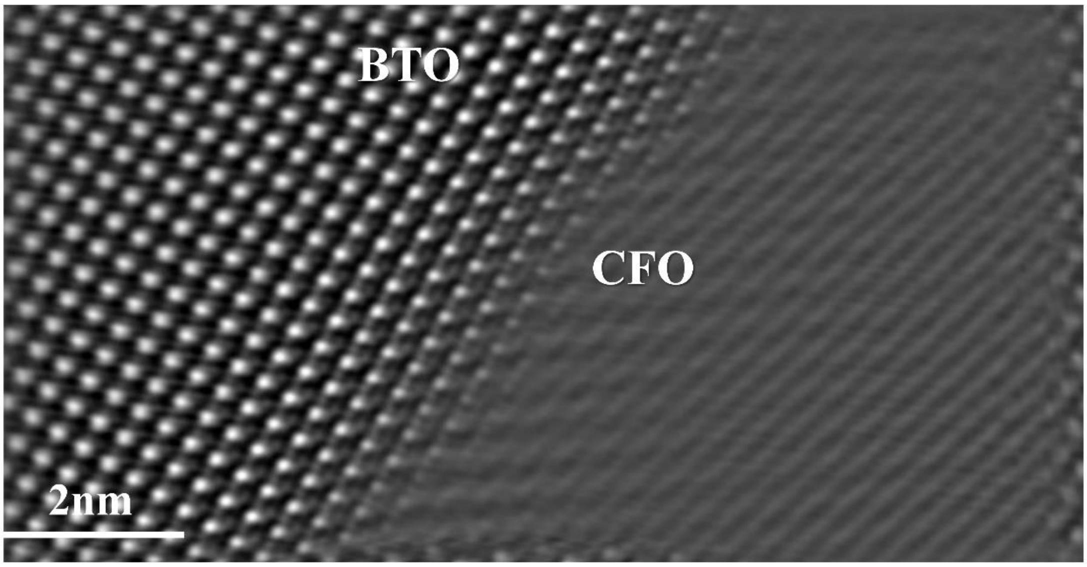

Fig. S3. Nanostructure under high-angle annular dark-field scanning transmission electron microscope. A clear interface between the two phases can be seen, but the atomic structure cannot be clearly seen in the right half, which is due to the height difference between the BTO and CFO phase. The height of CFO is higher than BTO, which can indicate that CFO grows on BTO.

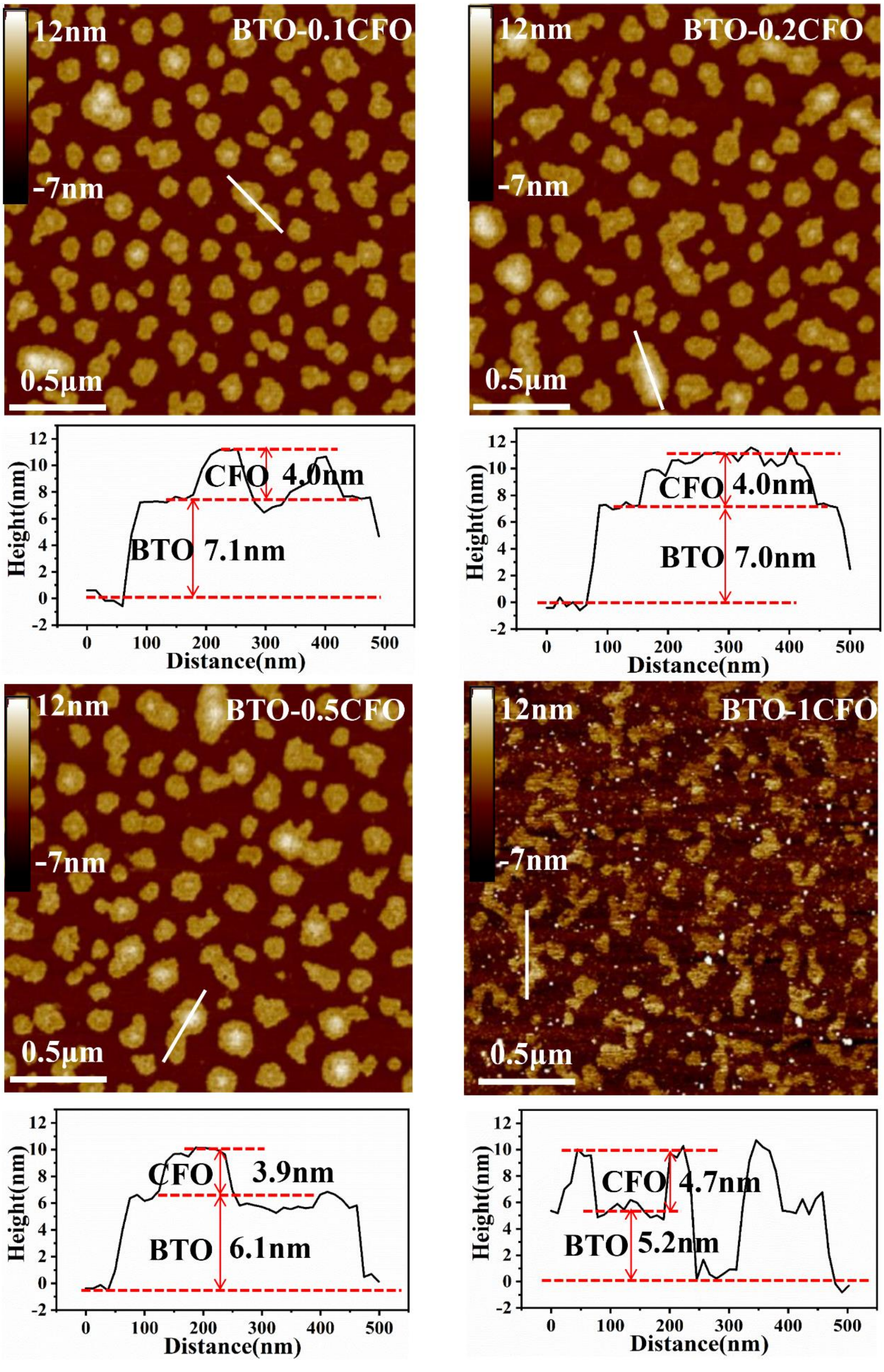

Fig. S4. AFM images of the four structures and the corresponding height measurements of the lines in the AFM image. The height information in the figure corresponds to the BTO and CFO thicknesses.

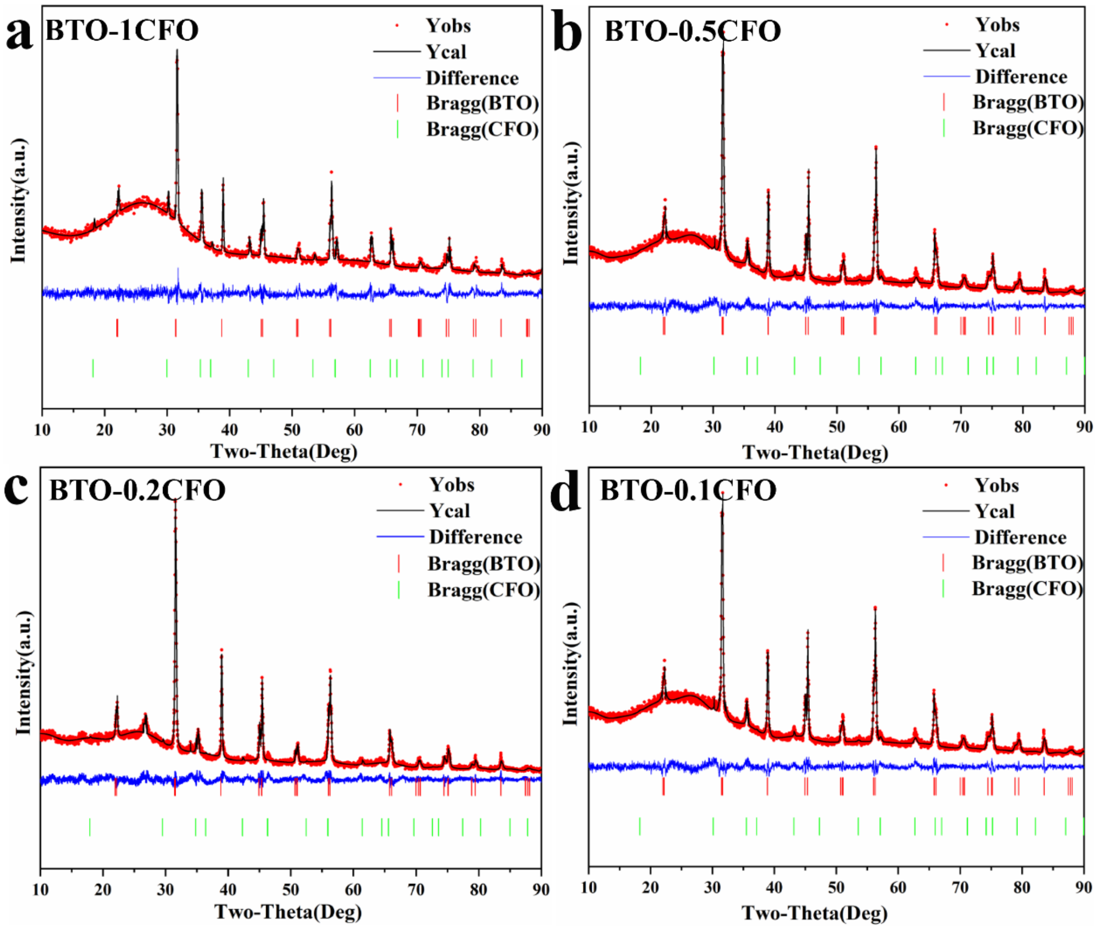

Fig. S5. X-ray diffraction refinement of the four structures.

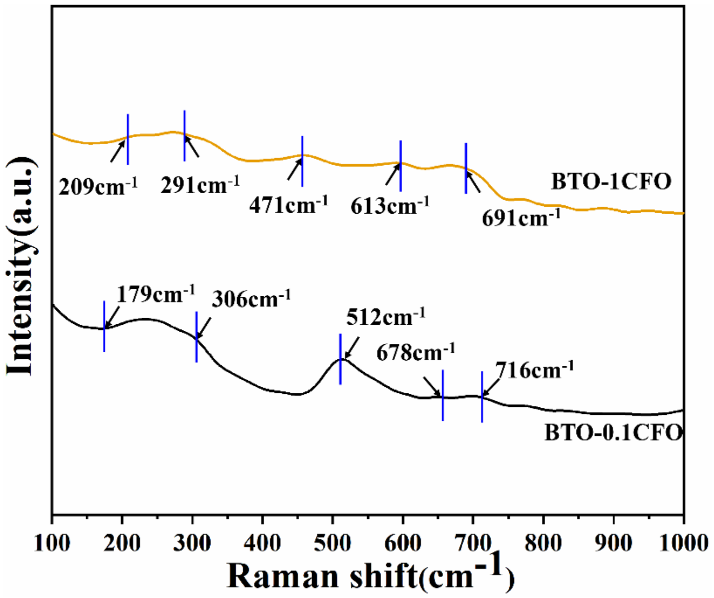

Fig. S6. Structural analysis by Raman spectroscopy.

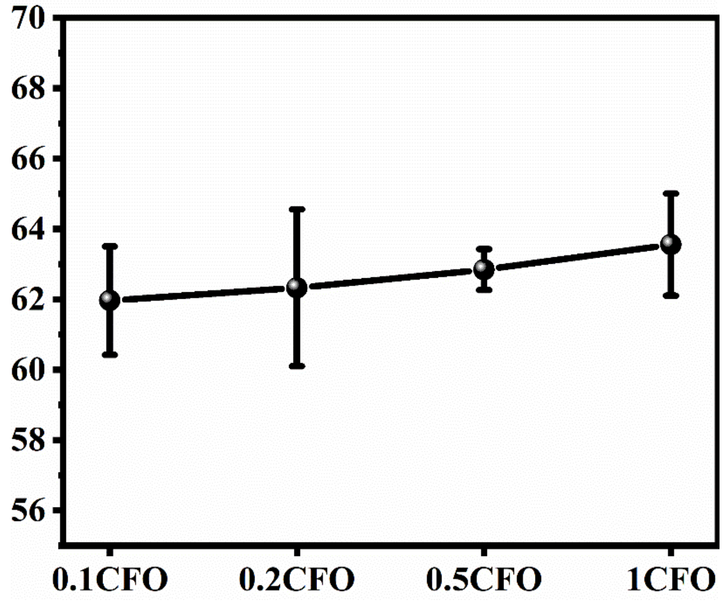

Fig. S7. Ms data obtained by normalizing the CFO content.

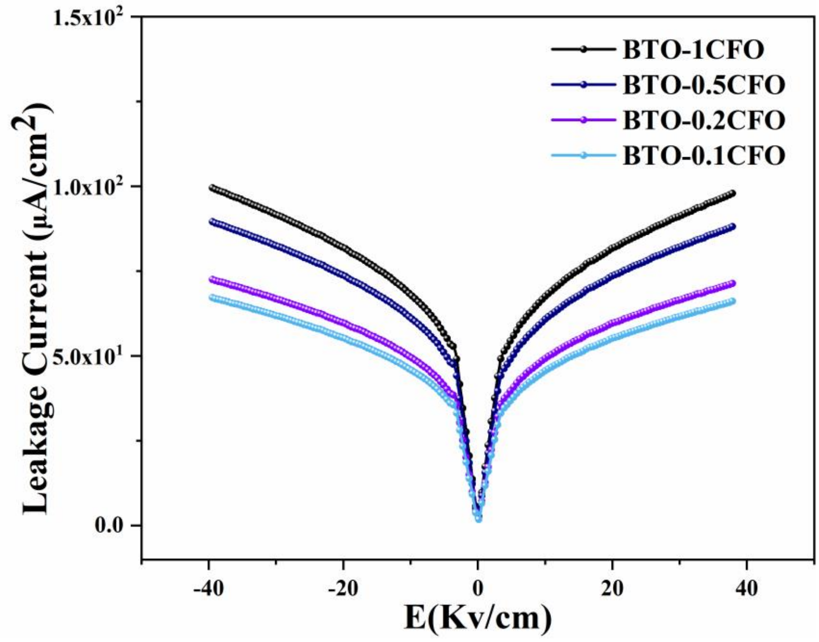

Fig. S8. Leakage current density (J) as a function of the electric field (E).

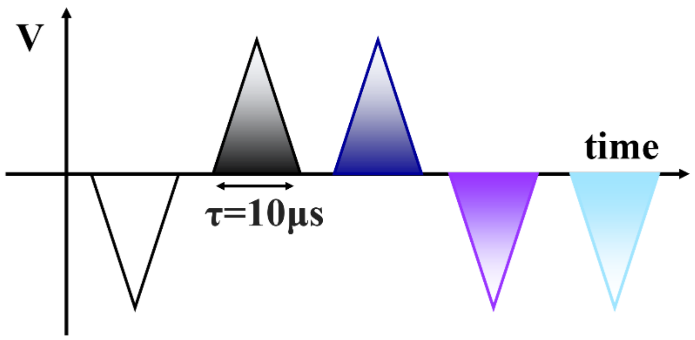

Fig. S9. Principle diagram of positive-up–negative-down (PUND) test (The pulse width is 10 μs and the delay between two pulses is 1 ms).

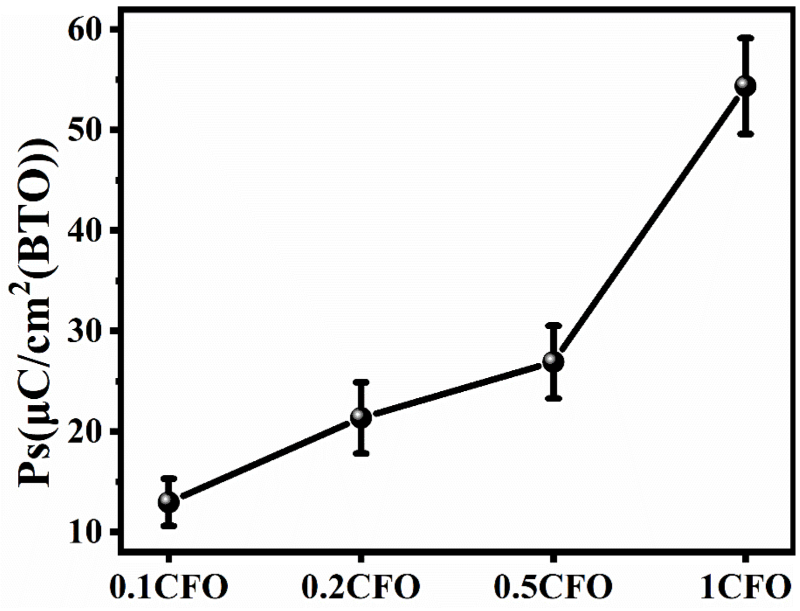

Fig. S10. Ps data obtained by normalizing the BTO content.

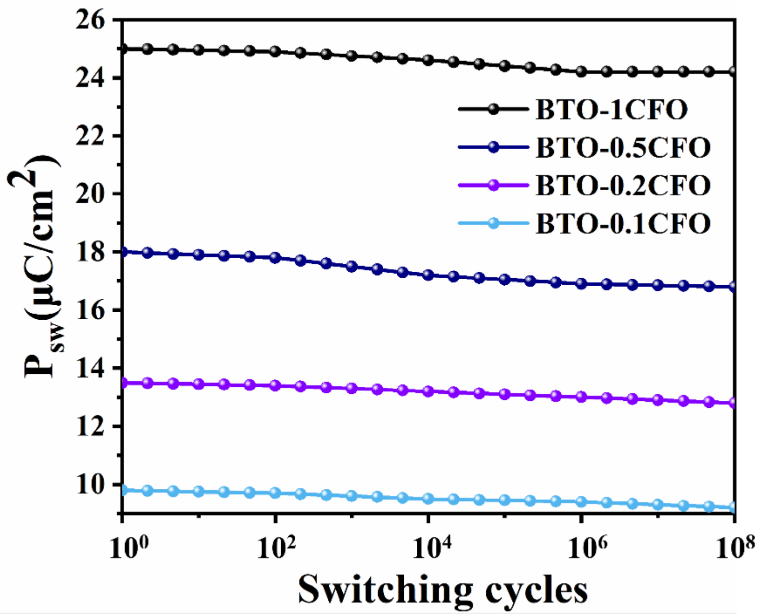

Fig. S11. Endurance properties of the samples.

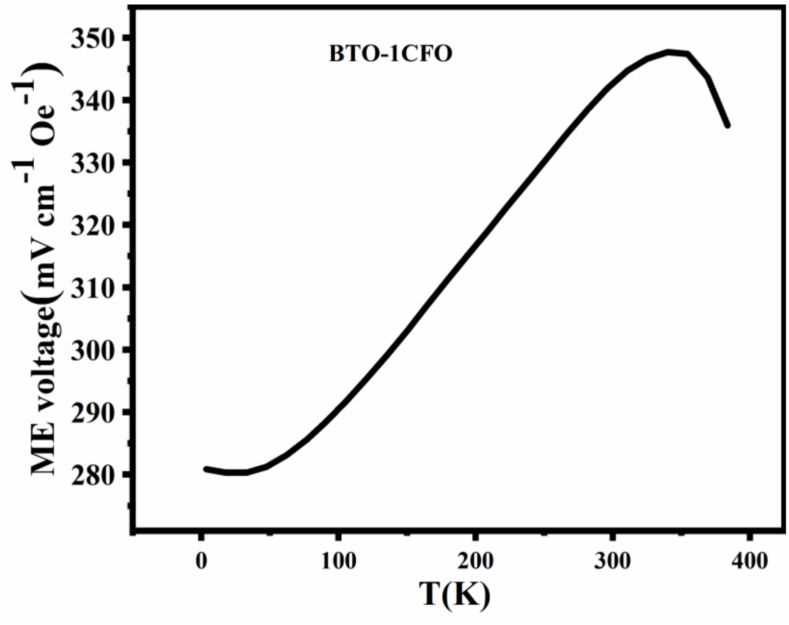

Fig. S12. Temperature-dependent ME response of BTO-1CFO at 1 kHz.

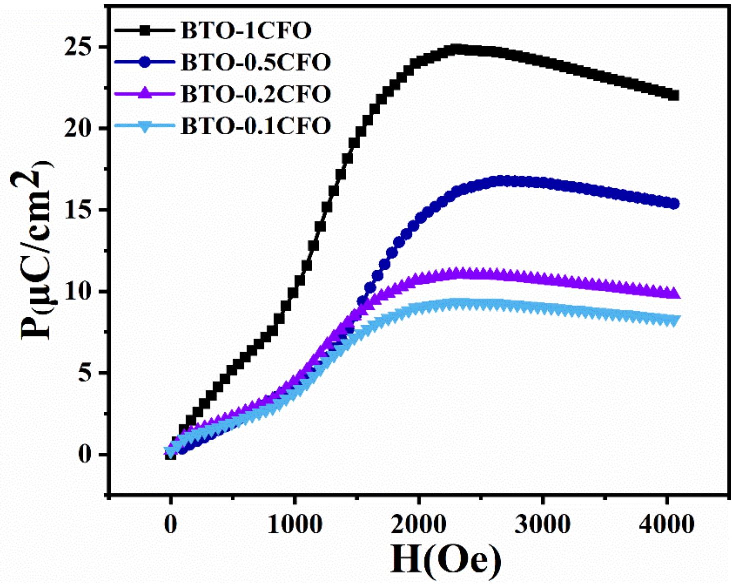

Fig. S13. Electric polarization as a function of magnetic field.

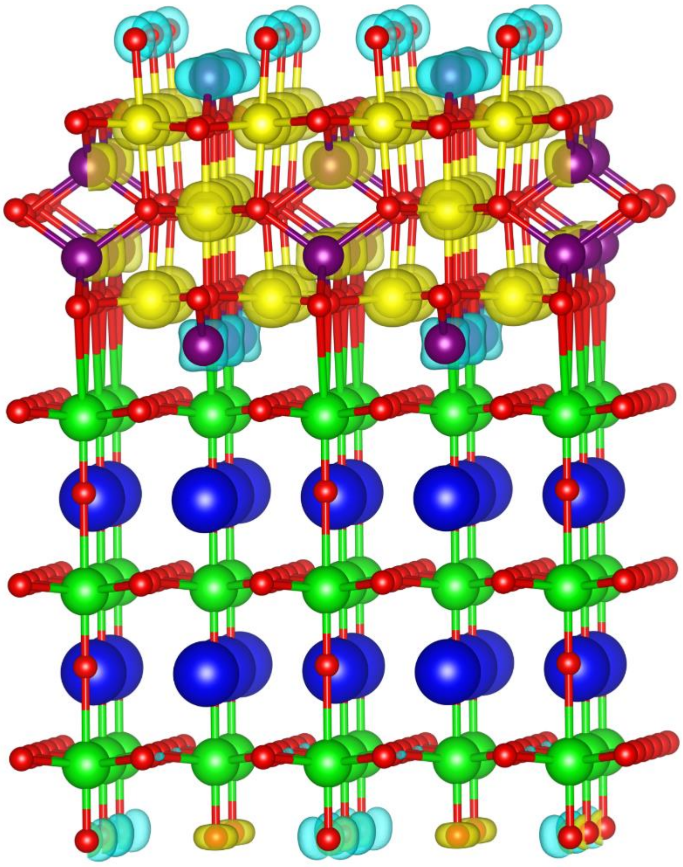

Fig. S14. Spin charge density distribution of BTO-CFO superlattice.

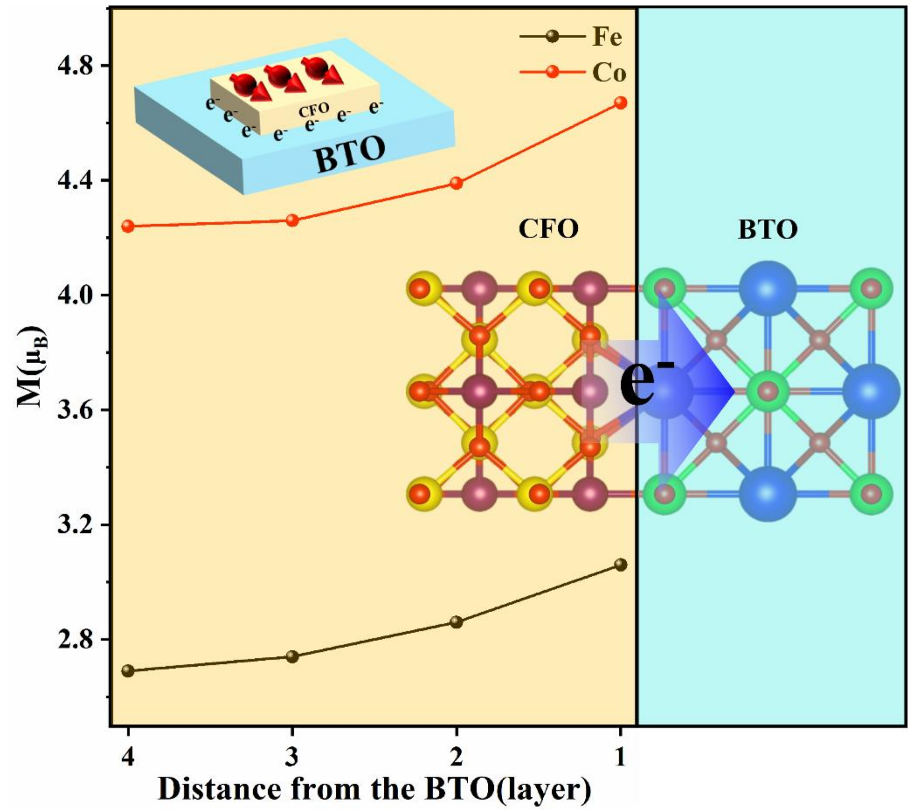

Fig. S15. Layer-by-layer Fe and Co atomic magnetic moments at the BTO-CFO interface.

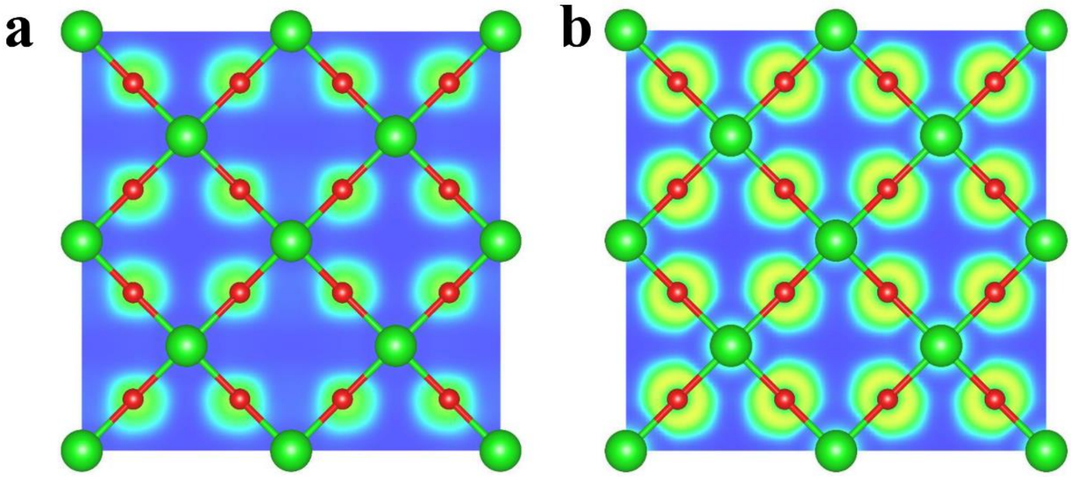

Fig. S16. Electron localization function at the BTO-CFO superlattice interface before (a) and after (b) optimization.

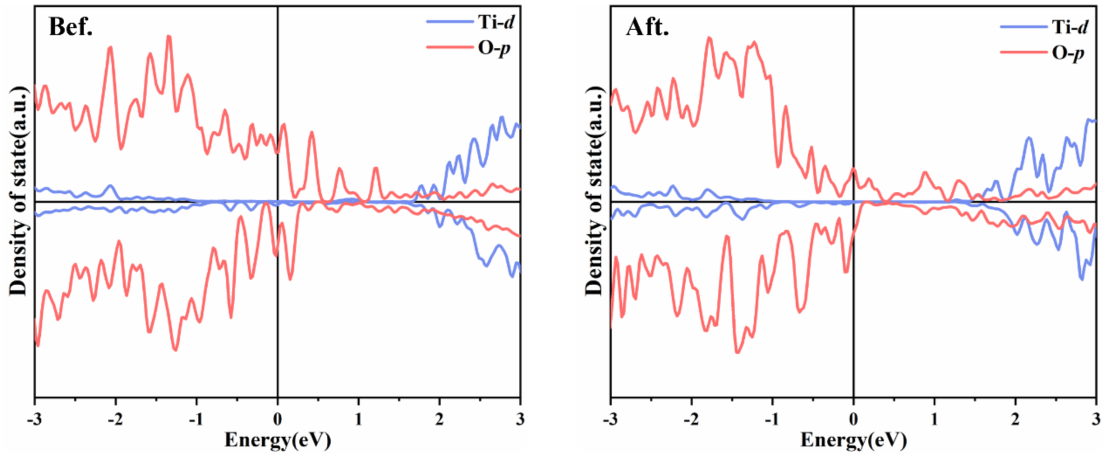

Fig. S17. Partial density of states (PDOS) at the BTO-CFO superlattice interface before (a) and after (b) structural optimization.

Supplementary Tables

Table S1. XRD refined fitting parameters and phase fractions.

Sample	Rp	Rwp	χ^2	Goodness of fit S	Phase ratio (BTO:CFO)
**BTO-0.1CFO**	4.62%	7.34%	2.22	1.49	1:0.14
**BTO-0.2CFO**	8.78%	5.34%	1.89	1.76	1:0.22
**BTO-0.5CFO**	5.61%	4.23%	1.91	1.88	1:0.42
**BTO-1CFO**	7.05%	5.37%	1.67	1.64	1:0.90

Table S2. Lattice parameters of BTO obtained by lattice XRD refinement.

Sample	a (Å)	c (Å)	c/a
**BTO-0.1CFO**	3.9858	4.0209	1.0088
**BTO-0.2CFO**	3.9831	4.0271	1.0110
**BTO-0.5CFO**	3.98171	4.0267	1.0113
**BTO-1CFO**	3.9861	4.1993	1.0535

Table S3. Saturated magnetization (Ms) values normalized to CFO content.

Sample	Ms(emu/g)	Phase ratio (BTO:CFO)	Ms(emu/g(CFO))
**BTO-0.1CFO**	7.61	1:0.14	61.97
**BTO-0.2CFO**	11.24	1:0.22	62.33

**BTO-0.5CFO**	18.59	1:0.42	62.85
**BTO-1CFO**	30.11	1:0.90	63.56

Table S4. Spontaneous polarization (Ps) values normalized to BTO content.

Sample	Ps(μC/cm^2)	Phase ratio (BTO:CFO)	Ps(μC/cm^2(BTO))
**BTO-0.1CFO**	11.37	1:0.14	12.96
**BTO-0.2CFO**	17.52	1:0.22	21.37
**BTO-0.5CFO**	18.95	1:0.42	26.91
**BTO-1CFO**	28.61	1:0.90	54.36

Table S5. Reported ME coupling coefficient in other ME composites.

ME composite	ME coefficient (mV cm^{-1} Oe^{-1})	Ref.
**$BaTiO_3$- $CoFe_2O_4$**	14	***J. Alloys Compd.*** 2019, 796, 55-64 [1]
**$BaTiO_3$- $CoFe_2O_4$**	12	***Vacuum*** 2022, 200, 110978 [2]
**$BaTiO_3$- $CoFe_2O_4$**	135	***J. Alloys Compd.*** 2015, 644, 390-397 [3]
**$BaTiO_3$- $CoFe_2O_4$**	42.57	***J. Magn. Magn. Mater.*** 2019, 469, 483-493 [4]
**$BaTiO_3$- $CoFe_2O_4$**	8	***Solid State Commun.*** 2012, 152, 1951-1955 [5]
**$BaTiO_3$- $CoFe_2O_4$**	3.88	***J. Magn. Magn. Mater.*** 2007, 310, e361–e363 [6]
**$BaTiO_3$- $CoFe_2O_4$**	2.8	***Physica B*** 2020, 599, 412577 [7]
**$BaTiO_3$- $CoFe_2O_4$**	50	***J. Mater. Sci.*** 1974, 9, 1705–1709 [8]
**(1-x)(BFO–BTO)/xCFO**	26.2	***Mater. Lett.*** 2016, 164, 388-392 [9]
**$NdMnO_3$- $BaTiO_3$**	22	***Materi. Chem. Phys.*** 2021, 270, 124856 [10]
**$PbZr_{0.52}Ti_{0.48}O_3$-$CoFe_2O_4$**	163	***J. Appl. Phys.*** 2006, 100, 094106 [11]
**$CoFe_2O_4$–$Pb(Fe_{1/2}Ta_{1/2})O_3$**	200	***Compos. Struct.*** 2010, 92, 2153-2158 [12]
**$CoFe_2O_4$–Pb(Zr,Ti)O_3**	92	***Phys. Rev. B*** 2007, 75, 174422 [13]
**$BaTiO_3$- $CuFe_2O_4$**	22.5	***J. Alloys Compd.*** 2018, 731, 288-296 [14]

Table S6. Polarization values along the Z-axis direction before and after structural optimization of BTO.

	**Polarization value (μC/cm2)**
**Before**	15.12
**After**	22.53

References

[1] A. Guzu, C. E. Ciomaga, M. Airimioaei, L. Padurariu, L. P. Curecheriu, I. Dumitru, F. Gheorghiu, G. Stoian, M. Grigoras, N. Lupu, M. Asandulesa, L. Mitoseriu, *J. Alloys. Compd.* **2019**, *796*, 55-64.

[2] X. Zhao, R. Cui, C. Deng, *Vacuum* **2022**, *200*, 110978.

[3] H. Yang, G. Zhang, Y. Lin, *J. Alloys. Compd.* **2015**, *644*, 390-397.

[4] K. C. Verma, M. Singh, R. K. Kotnala, N. Goyal, *J. Magn. Magn. Mater.* **2019**, *469*, 483-493.

[5] S. Agarwal, O. F. Caltun, K. Sreenivas, *Solid State Commun*. **2012**, *152*, 1951-1955.

[6] G. V. Duong, R. Groessinger, R. Sato Turtelli, *J. Magn. Magn. Mater.* **2007**, *310*, e361-e363.

[7] G. H. Rather, M. Ikram, *Physica B* **2020**, *599*, 412577.

[8] J. Van Den Boomgaard, D. R. Terrell, R. A. J. Born, H. F. J. I. Giller, *J. Mater. Sci.* **1974**, *9*, 1705-1709.

[9] H. Yang, G. Zhang, Y. Lin, *Mater.Lett.* **2016**, *164*, 388-392.

[10] S. Shankar, O. P. Thakur, M. Jayasimhadri, *Mater. Chem. Phys.* **2021**, *270*, 124856.

[11] J.-p. Zhou, H.-c. He, Z. Shi, G. Liu, C.-W. Nan, *J. Appl. Phys.* **2006**, *100*, 094106.

[12] J. Kulawik, P. Guzdek, D. Szwagierczak, A. Stoch, *Compos. Struct.* **2010**, *92*, 2153-2158.

[13] V. M. Petrov, G. Srinivasan, U. Laletsin, M. I. Bichurin, D. S. Tuskov, N. Paddubnaya, *Phys. Rev. B* **2007**, *75*, 174422.

[14] R. M. Thankachan, B. Raneesh, A. Mayeen, S. Karthika, S. Vivek, S. S. Nair, S. Thomas, N. Kalarikkal, *J. Alloys. Compd.* **2018**, *731*, 288-296.
